# Improved Algorithms for the Classification of Rough Rice Using a Bionic Electronic Nose Based on PCA and the Wilks Distribution

**DOI:** 10.3390/s140305486

**Published:** 2014-03-19

**Authors:** Sai Xu, Zhiyan Zhou, Huazhong Lu, Xiwen Luo, Yubin Lan

**Affiliations:** 1 Key Laboratory of Key Technology on Agricultural Machine and Equipment, South China Agricultural University, Ministry of Education, Guangzhou 510642, China; E-Mails: 294504658@qq.com (S.X.); huazlu@scau.edu.cn (H.L.); xwluo@scau.edu.cn (X.L.); 2 College of Engineering, South China Agricultural University, Guangzhou 510642, China; 3 United States Department of Agriculture, Agricultural Research Service (USDA-ARS), College Station, TX 77845, USA; E-Mail: yubin.lan@ars.usda.gov

**Keywords:** wilks distribution, principle component analysis (PCA), bionic electronic nose, gas sensor, rough rice, classification and recognition, probabilistic neural networks

## Abstract

Principal Component Analysis (PCA) is one of the main methods used for electronic nose pattern recognition. However, poor classification performance is common in classification and recognition when using regular PCA. This paper aims to improve the classification performance of regular PCA based on the existing Wilks Λ-statistic (*i.e.*, combined PCA with the Wilks distribution). The improved algorithms, which combine regular PCA with the Wilks Λ-statistic, were developed after analysing the functionality and defects of PCA. Verification tests were conducted using a PEN3 electronic nose. The collected samples consisted of the volatiles of six varieties of rough rice (Zhongxiang1, Xiangwan13, Yaopingxiang, WufengyouT025, Pin 36, and Youyou122), grown in same area and season. The first two principal components used as analysis vectors cannot perform the rough rice varieties classification task based on a regular PCA. Using the improved algorithms, which combine the regular PCA with the Wilks Λ-statistic, many different principal components were selected as analysis vectors. The set of data points of the Mahalanobis distance between each of the varieties of rough rice was selected to estimate the performance of the classification. The result illustrates that the rough rice varieties classification task is achieved well using the improved algorithm. A Probabilistic Neural Networks (PNN) was also established to test the effectiveness of the improved algorithms. The first two principal components (namely PC1 and PC2) and the first and fifth principal component (namely PC1 and PC5) were selected as the inputs of PNN for the classification of the six rough rice varieties. The results indicate that the classification accuracy based on the improved algorithm was improved by 6.67% compared to the results of the regular method. These results prove the effectiveness of using the Wilks Λ-statistic to improve the classification accuracy of the regular PCA approach. The results also indicate that the electronic nose provides a non-destructive and rapid classification method for rough rice.

## 1. Introduction

Classification and recognition has been widely used in various fields [1]. With the rapid development of sensor technology and computer technology, the use of a bionic electronic nose comprised of a semiconductor gas sensitive sensor and a pattern recognition system as a recognition tool provides a new method for rapid classification and recognition of items [2,3]. Rough rice is the first state of rice grains. Being wrapped in the hull makes rough rice barely recognisable by the eye. With the demands for improved rice grain quality, determining how to classify and recognise rough rice non-destructively and rapidly is a problem that researchers in this field strive to solve [4,5]. An electronic nose provides a new method to classify and recognise rough rice non-destructively and rapidly [6–8]. Pattern recognition methods include Principal Component Analysis (PCA) [9], Linear Discriminate Analysis (LDA) [10], Neural Networks (NNs) [11], *etc.* As a classical classification and recognition method, PCA is commonly used for electronic nose classification and recognition. Zheng *et al.* used an electronic nose (Cyranose-320, Cyranose Inc., Pasadena, CA, USA) to recognise four varieties of polished rice: Mahatma Brown Rice (MB), Riceland Milled Rice (RL), Thailand Jasmine Rice (TH) and Zatarain's Parboiled Rice (PR). Their study indicated the possibility of rice recognition using an electronic nose, but they mentioned that the classification and recognition effect could not reach the ideal situation when using PCA, as the method grouped PR with three other rice varieties that cannot be classified with each other [7]. Hu *et al.* used an electronic nose (PEN2, Airsense Analytics GmbH, Schwerin, Germany) for the detection of volatiles and the variety recognition of aromatic rice (Tiandongxiang, Exiang 1) and non-aromatic rice (Zheyou 1, Kehan1 and Liangyoupeijiu). The result indicated that polished rice has the best recognition effect, with all of the rough rice varieties being recognised except for Liangyoupeijiu and Zheyou 1 rough rice, which have overlaps; the recognition effect of five cooked rice and brown rice varieties was the worst when PCA was used for the analysis [8]. Yu *et al.* used an electronic nose for the recognition of four rice grain varieties growing in the same area. The paper also mentioned that Fengliangyou 4 has a large overlap with Zajiao 838 and could not be classified [6].

Principal Component Analysis is usually chosen as the first and second principal component (PC) according to the cumulative sensor contributions when using PCA. However, PCA often cannot produce the best recognition effect when using the first and second principal components for PCA. For this purpose, the Wilks distribution [12] helps provide a new way and method for choosing principal components when using PCA for analysis. Yin *et al.* used a method that combines PCA with the Wilks distribution to successfully recognise three types of Chinese drinks. The result indicated that the recognition effect using PC4 and PC5 is better than that using PC1 and PC2 [13]. Yin *et al.* provided a further analysis of the reason why the three Chinese drinks recognition using PC4 and PC5 is better than that using PC1 and PC2. Their loading plots indicated that the points plotted using PC1 loading and PC2 loading are rather close together, being only in a small area apart from one point, so that the information given by PC1 and PC2 may fall into the same category and cannot reflect the features of broad-spectrum caused by cross-sensitivity reactivity. In addition, the information given by PC4 and PC5 is not so strong, but the information is richer and may reflect the broad-spectrum features [14]. Zhou *et al.* used a method that combines PCA with the Wilks distribution to successfully recognise two types of ginseng antler strength wine. The results show that the recognition effect by PC2 and PC7 is better than that by PC1 and PC2 [15].

In the process of the classification and recognition of hybrid and inbred rough rice varieties, we also met the difficulty that the recognition effect of PCA cannot reach the ideal state. This paper aims to analyse the problem of the existing combination of PCA with the Wilks distribution method, determine an improved method, classify and recognise rough rice varieties and use the Mahalanobis Distance (MD) and Probabilistic Neural Networks (PNN) to verify the method. This paper also proposes a new method for rough rice classification and recognition.

## 2. Materials and Methods

### 2.1. Preparation of Samples

**Figure 1.**
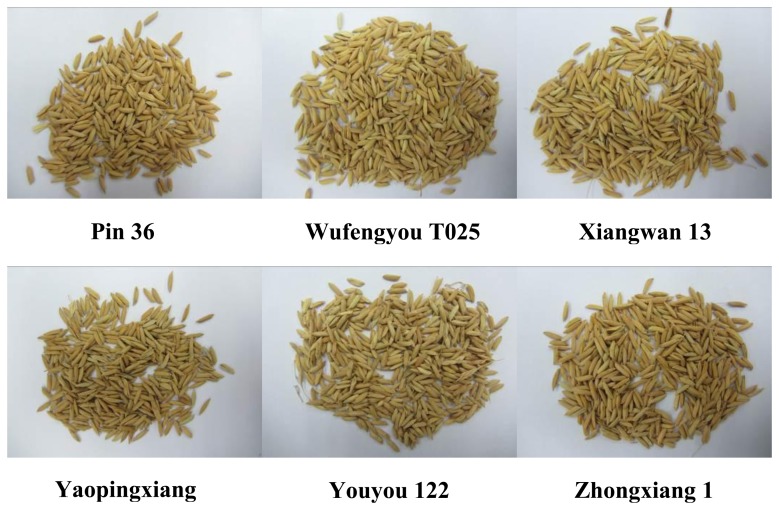
The six studied varieties of rough rice.

The six types of rough rice varieties selected in this experiment were planted on the farm (Yuejinbei) of South China Agricultural University. They included three inbred rough rice varieties (Zhongxiang1, Xiangwan13, Yaopingxiang) and three hybrid rough rice varieties (WufengyouT025, Pin 36, Youyou122). These varieties have the same crops for rotation. The harvest time differences among them do not surpass 30 days. After harvest, natural drying to keep the water content between 12%–14% via the method of sunning on cement ground was performed. The characteristic appearance of the six types of rough rice is shown in Figure 1.

### 2.2. Electronic Nose Set-Up

**Figure 2.**
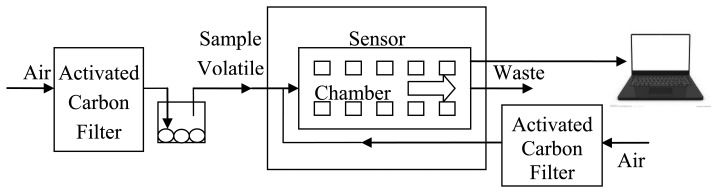
The structure of the portable electronic nose.

A portable electronic nose (PEN3, Airsense Analytics GmbH) is used in this experiment. This electronic nose is mainly composed of a sensor array, sampling and cleaning channel, data processing system, *etc.* The system structure is shown in Figure 2. The sensor array is composed of 10 metal-oxide sensors, which are the core components of the electronic nose. Each sensor is sensitive to different volatiles. The ith (from 1 to 10) sensor's response data R_i_ is the ratio of the resistance value G (when sensors contact to sample volatiles) and the resistance value G_0_ (when sensors contact to zero gas).

The zero gas used by the PEN3 is the field air, which is filtered by an activated carbon filter. The special flow regulator inside can guarantee stable sampling under poor experiment conditions. The detection principle is as follows: when volatile compounds contact the active material of the sensor, it will create a transient response (a series of physical and chemical changes occur). This response from the voltage signal translates into the figure signal via an interface circuit, which is then recorded via a computer and sent to a signal processing unit for analysis. Afterwards, a comparison is made with a large number of volatile compound information in a database that can compare and identify the type of volatiles [16,17].

**Figure 3.**
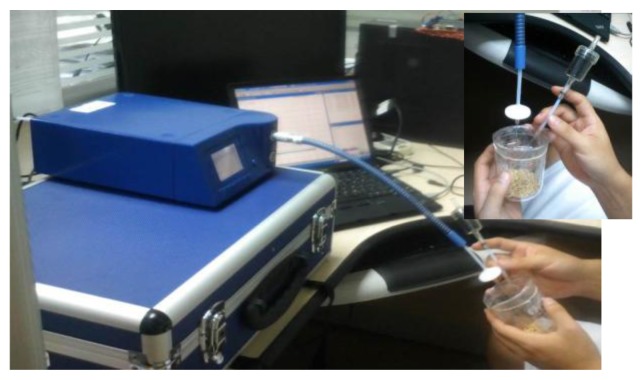
Sampling set-up using the electronic nose.

Figure 3 shows the sampling set-up for the six types of rough rice.

### 2.3. Pre-Run Procedures and Data Collection

There were 20 samples of each rough rice variety (6 varieties of rough rice × 20 = 120 samples in total). Each sample weighed 10 g, measured using an electronic scale, and was collected in a 200-mL beaker, then sealed with plastic wrap. Before sampling, every sample was kept at room temperature environment (27 °C) for 1 h. Beakers were washed using an ultrasonic cleaning instrument and cooled in the shade, and no peculiar smell was detected. Preheating for 10 min before the measurement was performed to ensure that the sensors reach their working temperature. Zero gas was used to flush the induction trunk of the electronic nose before sampling. The working parameter settings are as follows: sampling interval is 1 s; flush time is 60 s; zero point trim time is 10 s; measurement time is 80 s; presampling time is 5 s; and injection flow is 190 mL/min.

### 2.4. Feature Extraction

**Figure 4.**
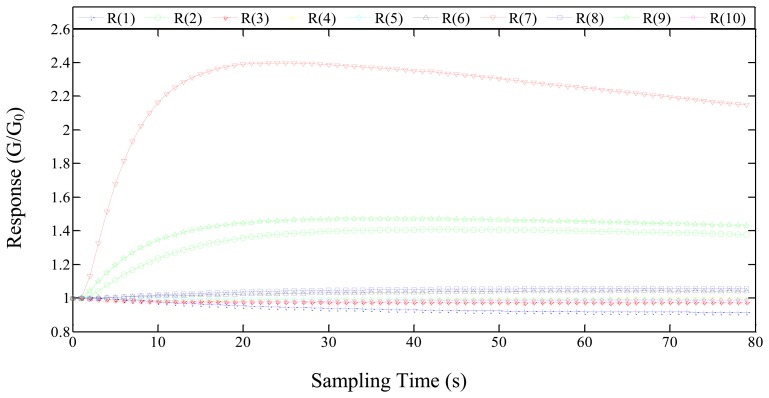
The electrical signal change in volatile detection of “Youyou 122” rice grain sample (where R(1)–R(10) are the numbers of the 10 metal-oxide sensors in the sensor array).

Figure 4 shows the response of the electronic nose to the “Youyou122” rice grain sample. The feature extraction should contain as much feature information as possible. The mean-differential coefficient value can reflect the average velocity of the response of the sensor and represent its major features [18]. Thus, we choose the mean-differential coefficient value (D_ave_) as the feature value of response curve of the sensor. The test results constitute a 120 (120 samples in total) × 10 (10 sensors) matrix. The D_ave_ is defined as follows:

(1) $$D_{ave}=\frac{1}{n-1}\sum_{z=1}^{n-1}\frac{{\text{x}}_{z+1}-x_{z}}{\Delta t}$$

where n is the total tests (n = 80) of a sensor to a sample, *x_z_* is the zth text result of a sample, *x_z_*_+_*_1_* is the (z + 1) th text result of a sample, *Δt* is the time interval (*Δt* = 1 s) of two neighbourhood text results.

### 2.5. Improved Algorithms Combining Regular PCA with the Wilks Λ-statistic

#### 2.5.1. Principle of the Wilks Λ-Statistic

PCA is a multivariate technique that analyses a data table in which the observations are described by several inter-correlated quantitative dependent variables. The goal of PCA is to extract the important information from the table, to represent it as a set of new orthogonal variables called principal components, and to display the pattern of similarity of the observations and of the variables as points in maps [19]. The Wilks Λ-statistic is typically used to test or examine the differences between two or more populations [20]. The PCA combined with the Wilks Λ-statistic is a new method that can provide the best way to choose principle components for PCA. We can achieve an advanced selection method of the principal components by using the Wilks Λ-statistic for improving the classification effect of six types of rough rice. The relevant mathematical calculations that were developed by Yin [13,14] and Zhou [15] are as follows: regarding the variables, m is the number of PCs that are selected, *D* is the matrix of the sum of the squares of the deviations within classes, and *A* is the matrix of the total sum of the squares of the deviations for classes. *D* and *A* are given by:

(2) $$D={\left(d_{ij}\right)}_{m\times m}$$

(3) $$A={\left(a_{ij}\right)}_{m\times m}$$

(4) $$d_{ij}=\sum_{g=1}^{c}\sum_{k=1}^{N_{g}}\left(X_{igk}-u_{ig})*(X_{jgk}-u_{jg}\right)$$

(5) $$a_{ij}=\sum_{g=1}^{c}\sum_{k=1}^{N_{g}}\left(X_{igk}-u_{i})*(X_{jgk}-u_{j}\right)$$

where c is the number of classes (c = 6) corresponding to six types of rough rice, N_g_ is the sample number of the gth class (N_1_ = N_2_ = N_3_ = N_4_ = N_5_ = N_6_ = 20), *u_ig_* is the average corresponding the ith PC of the gth class, and u_i_ is the average corresponding to the ith PC of the total classes.

According to the idea of Wilks distribution, the lower the value of |D| and the higher the value of |A| and the more significant is the difference between classes; it is useful to classify these classes. Λ is the Wilks Λ-statistic, defined as:

(6) $$\Lambda =\frac{\left|\text{D}\right|}{\left|\text{A}\right|}$$

##### 2.5.2. Improved Algorithms Based on PCA and the Wilks Λ-Statistic

In actual operation, the deviation values *X_igk_-u_ig_*, *X_jgk_-u_jg_*, *X_igk_-u_i_* and *X_jgk_-u_j_* of each element in any PC may be opposite in sign, thus causing the products of the deviation values (*X_igk_* - *u_ig_*) × (*X_jgk_* - *u_jg_*) or (*X_igk_* - *u_i_*) × (*X_jgk_* - *u_j_*) in any two PCs to be opposite in sign too. This behaviour may make the products of the deviation values (*X_igk_* - *u_ig_*) × (*X_jgk_* - *u_jg_*) or (*X_igk_* - *u_i_*) × (*X_jgk_* - *u_j_*) cancel out when in the summation operator. This phenomenon is contrary to the purpose of the summation operator.

**Figure 5.**
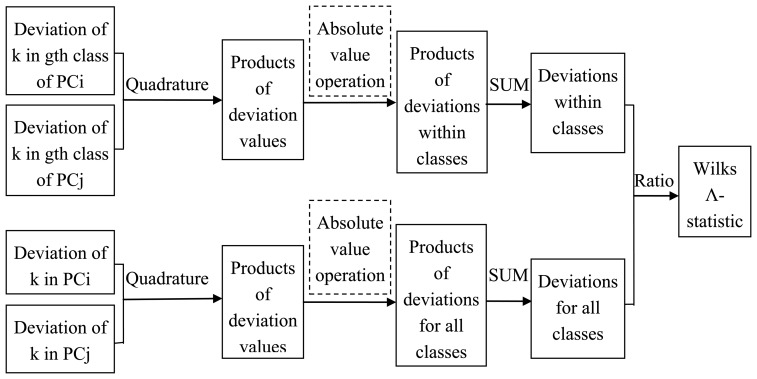
The flow diagram of the improved algorithm.

For this situation, this paper proposes that after getting the products of the deviation values of (*X_igk_* − *u_ig_*) × (*X_jgk_* − *u_jg_*) or (*X_igk_* − *u_i_*) × (*X_jgk_* − *u_j_*), one should take the absolute value first, then use the summation operator to calculate the deviation values. And the other operation steps remain unchanged. The improved algorithms are as follows. The flow diagram for the improved algorithms is shown in Figure 5. The operation steps in the dashed box are added for the improved algorithms.

(7) $$d_{ij}=\sum_{g=1}^{c}\sum_{k=1}^{N_{g}}\left|\left(X_{igk}-u_{ig})\times (X_{jgk}-u_{jg}\right)\right|$$

(8) $$a_{ij}=\sum_{g=1}^{c}\sum_{k=1}^{N_{g}}\left|\left(X_{igk}-u_{i})\times (X_{jgk}-u_{j}\right)\right|$$

#### 2.6. Establishment of the PNN Model

**Figure 6.**
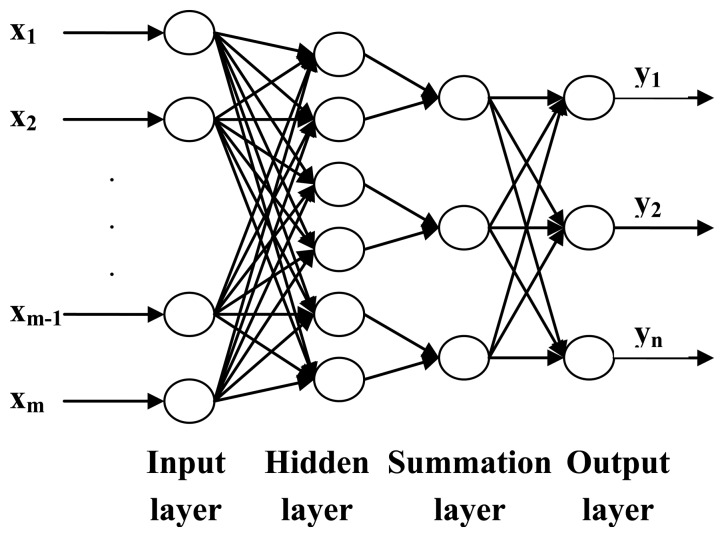
The diagram of the PNN.

A Probabilistic Neural Network (PNN) is a type of neural network with a simple construction and wide application that was developed by Specht in 1989 [21]. The use of a PNN can achieve high accuracy by replacing a nonlinear algorithm with a linear algorithm and is widely applied in pattern classification. PNN includes an input layer, a hidden layer, a summation layer and an output layer [22]. The network structure of a PNN is shown in Figure 6 [23,24]. The first layer is the input layer, which used to receive the values of the test samples. The first layer is functionally a linear algorithm, and the number of neurons in the layer is equal to the number of the inputs. The second layer is a hidden layer, which connected with the input layer by weight W_ij_. The transfer function of the second layer is g(z_i_) = exp((z_i_ − 1)/σ^2^), where z_i_ is the input value of the i-th neuron, and σ is the average variance. The third layer is the summation layer, which has the function of linear summation. The amount of neurons of the third layer is equal to the pattern number that is planned to be allocated. The last layer is the output layer, which has a judgment function. The outputs of this layer are discrete values 1 and −1 (or 0), which represent the respective classes of the input pattern [25].

### 3. Results and Discussion

#### 3.1. Regular PCA for the Classification of 6 Varieties of Rough Rice

**Figure 7.**
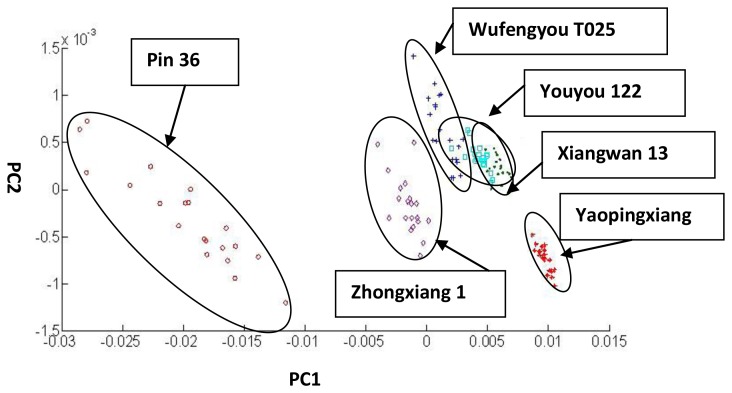
Classification of the six rough rice varieties using PC1 and PC2.

According to regular PCA, we can determine the feature values of each PC that were, from large to small, as follows: λ_1_ = 9.460 × 10^−5^, λ_2_ = 2.9085 × 10^−7^, λ_3_ = 8.0030 × 10^−8^, λ_4_ = 3.8928 × 10^−8^, λ_5_ = 1.9657 × 10^−8^, λ_6_ = 3.7732 × 10^−9^, λ_7_ = 8.2980 × 10^−10^, λ_8_ = 4.9310 × 10^−10^, λ_9_ = 1.8386 × 10^−10^, and λ_10_ = 8.4961 × 10^−11^. Then, λ_1_ and λ_2_ are chosen as PC1 and PC2 to perform the PCA. The amount of the variance accounted for by PC1 and PC2 was 99.85%. The classification result of PCA is shown in Figure 7. In this Figure, Wufengyou T025 is overlapping Youyou 122 and Youyou 122 is overlapping Xiangwan 13. Those three rough rice varieties cannot be classified using regular PCA.

#### 3.2. Improved Algorithms for the Classification of Six Rice Rough Varieties

**Figure 8.**
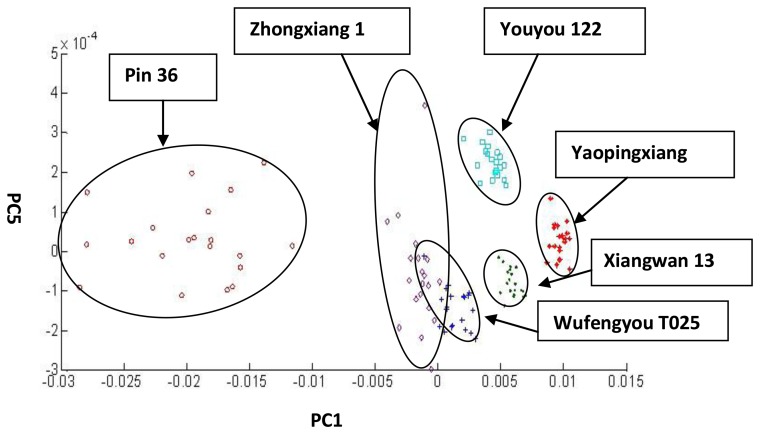
Classification of the six rough rice varieties using PC1 and PC5.

The results of the dispersion ratio when any two eigenvectors comprise a lower-dimensional matrix are shown in Table 1. The dispersion ratio of PC1 and PC5 was the smallest. According to the Wilks Λ-statistic, choosing PC1 and PC5 for the PCA can result in the best classification result. The amount of variance accounted for by PC1 and PC5 was 99.56%. The classification result of PC1 and PC5 is shown in Figure 8. All of the rough rice varieties can be classified, except for the overlap of Wufengyou T025 with Zhongxiang 1. Compared with the regular PCA classification result, which had three rough rice varieties that could not be classified, there were only two rough rice varieties that could not be classified using the Wilks Λ-statistic. This result proves that the Wilks Λ-statistic can effectively improve the classification accuracy of regular PCA.

#### 3.3. Comparison of the Mahalanobis Distance of the Classification Based on the Different PCs

The Mahalanobis distance (MD) is a commonly used distance detection method that can compute data correlations [26]. To study the suitability of the used Wilks Λ-statistic to improve the classification result of regular PCA, this paper used the MDs of each of the centre points of two sample data points (*x_ave_*,*y_ave_*) by two choices of PCs for comparison. The MDs of the centre points of each of the two sample data points is bigger, and the classification result is thus better. The comparison results are listed in Table 2.

In this table, choosing PC1 and PC5 for analysis, compared with choosing PC1 and PC2, the MDs of each of the centre points of two sample data points were enlarged: Xiangwan 13 and Pin 36, Pin 36 and Youyou 122, Youyou122 and Wufengyou T025, Youyou122 and Xiangwan 13, Zhongxiang 1 and Pin 36. These results are the same as the results of Figure 5 and Figure 6 and further verified that the Wilks Λ-statistic can effectively improve the classification accuracy of regular PCA.

(9) $$x_{ave}=\frac{\overset{20}{\underset{i=1}{\text{∑}}}x_{i}}{20}$$

(10) $$y_{ave}=\frac{\overset{20}{\underset{i=1}{\text{∑}}}y_{i}}{20}$$

where *x_i_* and *y_i_* are the *i*-th sample's value of a rough rice variety in one of two different PCs.

#### 3.4. Comparison of the PNN Classification Based on the Difference of the PCs

To further prove the suitability of the Wilks Λ-statistic, we use PC1 and PC2 and PC1 and PC5 as the inputs of the PNN for the respective classification of the six rough rice varieties. We choose the first 15 samples of each variety as the training samples and the remaining five samples as the test set. There are 90 training samples and 30 test samples in total. There are two neurons in the input layer, 120 neurons in the hidden layer, six neurons in summation layer and six neurons in output layer.

**Figure 9.**
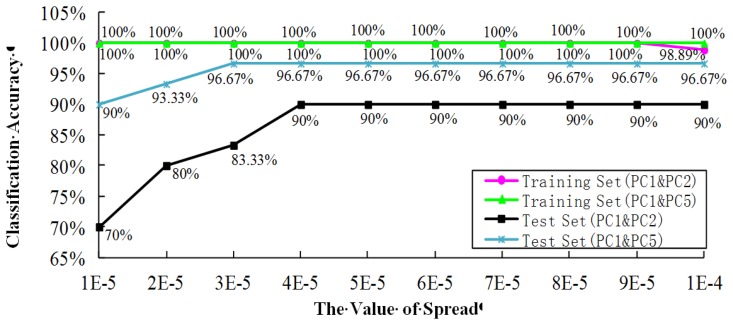
The selection of the spread value for a PNN.

This research used the “newpnn” function command of Matlab to develop the PNN model. Both PCs and the spread value have a certain influence to the classification results. The spread value is the diffusion rate of the PNN model that can be optimised for maximum classification accuracy, and its default is 0.1 [27]. If the spread value levels off to 0, the PNN model amounts to the nearest neighbour classifier. To optimise the PNN model, this research set the optimal range of the spread value to [1 × 10^−5^, 2 × 10^−5^, 3 × 10^−5^, 4 × 10^−5^, 5 × 10^−5^, 6 × 10^−5^, 7 × 10^−5^, 8 × 10^−5^, 9 × 10^−5^, 1 × 10^−4^]. The optimal results are shown in Figure 9.

As can been seen from the figure, comparing with PC1 and PC2, the classification accuracy of the test set based on PC1 and PC5 was improved 20% when spread = 1 × 10^−5^, which was improved 13.33% respectively when spread = 2 × 10^−5^ and 3 × 10^−5^.We chose the best model when both the classification accuracy of the training set and the test set were the highest at the same time. According to Figure 8, the PNN is the best when spread = [4 × 10^−5^, 5 × 10^−5^, 6 × 10^−5^, 7 ×10^−5^, 8 × 10^−5^, 9 × 10^−5^, 1 × 10^−4^], so we can set the best PNN to be the model by select the spread = 4 × 10^−5^. The best results were found when PC1 and PC5 were used for PCA, which increased the classification accuracy of PC1 & PC2 used for PCA by 6.67%, thereby proving the effectiveness of the Wilks Λ-statistic used for improving the classification accuracy of regular PCA. The classification results are described in Tables 3 and 4.

#### 3.5. Discussion

The purpose of this study was to determine a better method than conventional PCA to improve the classification accuracy of various rough rice varieties. The data analyses and results mentioned above provide demonstrative evidence of the effectiveness of using a combination of PCA with the Wilks distribution method.

More than 126 chemical species have been reported in volatile compounds released by various rough rice varieties, including hexanal, enanthal, nonanal, pentanal, isobutyl aldehyde, methanol, ethanol, acetonum, 4-vinylphenol, 2-pentylfuran, *etc*. [28]. The amount of each chemical component has a significant influence on the odor of rice varieties, thereby affecting the response of the sensor array of an electronic nose, so both the selectivity and the multi-functions of the gas sensor are very important facts to establish a volatiles-sensitive gas sensor array [29]. Typically, there are some relationships and differences among the gas sensors in the sensor array when it is used to distinguish odor samples. In another words, it shows some correlation, so we can realize the identification of various rough rice varieties based on an electronic nose.

The conventional PCA is a mathematical dimensionality reduction method. In general, it will find several aggregate variables which contain almost all the information in the original variables but no correlations with each other, to replace numerous original variables. It is inconsistent with the use of the overlap effect of the electronic nose. Especially when the difference between odor samples is small, the overlap effect among the sensor array is stronger than usual, and it is often more difficult for the conventional PCA method to conduct effective sample classification.

The improved algorithm presented in this study, which combines PCA and the Wilks Distribution (Wilks Λ-statistic), selects PCs by estimating the smallest ratio of D (deviations within classes) to A (deviations for all classes) for further PCA classification. It could maximize the correlation between eigenvectors, but minimize the correlation between eigenvalues. That is why the improved algorithms in this study can obtain better classification accuracy.

The capabilities established in this study demonstrate the tentative feasibility of using electronic noses for the classification of various rough rice varieties. However, there are still a number of potential problems associated with the application of electric noses for the classification of various rough rice varieties. Firstly, due to the sensitivities of gas sensors to humidity and temperature, the variability of humidity and temperature in the test environment can greatly affect the outputs of electronic noses. Following additional research to solve this problem some humidity and temperature compensation algorithms should be included. Secondly, the possibility of several rough rice varieties being mixed together may further complicate classification of rough rice varieties. In addition, there is also a need to reduce the number of sensors in the sensor array in order to reduce the cost of the electronic noses. The proper sensor design choices to achieve sensor sensitivity specific to classify rough rice varieties should help further optimize the number of sensors used in a sensor array. What's more, software improvements will seemingly resolve some of the problems.

### 4. Conclusions

PCA is one of the main methods for electronic nose pattern recognition. This paper aimed to understand why the classification effect of the first two PCs used for PCA is poor. To study this effect, a method that combines PCA with the Wilks distribution (Wilks Λ-statistic) was used to improve the classification accuracy of regular PCA. First, the functionality and defects of the Wilks Λ-statistic were analysed, which led to the development of improved algorithms.

Subsequently, the Wilks Λ-statistic was used for the classification of six rough rice varieties and then compared with the regular PCA classification result. The results indicated that there are three rough rice varieties that cannot be classified using the regular PCA and two rough rice varieties that cannot be classified using the Wilks Λ-statistic. A preliminary judgement was made that the classification effect of the Wilks Λ-statistic is better than that of the PCA. Next, the MDs of each of the centre points of two sample data points (*x_ave_*,*y_ave_*) from two choices of PCs were determined for comparison. The results demonstrated that the Wilks Λ-statistic solved the problem that Wufengyou T025, Youyou 122 and Xiangwan 13 could not be recognised by using the regular PCA, thereby further illustrating the effectiveness of the use of the Wilks Λ-statistic to improve the classification accuracy of regular PCA. The proposed Wilks Λ-statistic to improve the classification accuracy of indistinguishable samples is based on the decrease of the classification accuracy of distinguishable samples.

Finally, PC1, PC2 and PC1, PC5 were used as the inputs of a PNN for the respective classification of 6 rough rice varieties. Comparing with PC1 and PC2, the results showed that the classification accuracy of the test set based on PC1 and PC5 was improved 20% when *spread* = 1 × 10^−5^, which was improved 13.33% respectively when *spread* = 2 × 10^−5^ and 3 × 10^−5^. The PNN is the best when *spread* = [4 × 10^−5^, 5 × 10^−5^, 6 × 10^−5^, 7 × 10^−5^, 8 × 10^−5^, 9 × 10^−5^, 1 × 10^−4^]. We set the best PNN in this research as the model by select the spread = 4 × 10^−5^. The best results indicated that the use of PC1 and PC5 for PCA increased the classification accuracy compared to the use of PC1 and PC2 by 6.67%, thereby proving the effectiveness of the use of the Wilks Λ-statistic to improve the classification accuracy of regular PCA. In addition, this research provides a novel non-destructive and rapid classification method for rough rice electronic-nose classification that has a certain guiding significance.

## Figures and Tables

**Table 1.** The dispersion ratio when any two eigenvectors comprise a lower-dimensional matrix.

**PC**	**2**	**3**	**4**	**5**	**6**	**7**	**8**	**9**	**10**
1	0.1441	0.1855	0.1687	0.1254	0.1709	0.1877	0.1552	0.1407	0.1569
2		0.5154	0.4351	0.3127	0.3563	0.4921	0.4793	0.4642	0.4855
3			0.6940	0.5111	0.5122	0.7981	0.7470	0.7650	0.7846
4				0.4195	0.5632	0.6848	0.7219	0.7435	0.7360
5					0.3298	0.4631	0.4728	0.4979	0.5204
6						0.7126	0.6931	0.7189	0.6781
7							0.9207	0.9848	0.8886
8								0.9332	0.9511
9									0.9562

**Table 2.** MDs of the centre points of the sample data points for the six rough rice varieties.

			**Wufengyou T025**	**Xiangwan 13**	**Yaopingxiang**	**Youyou 122**	**Zhangxiang 1**
Pin36	PCs	1,2	2.6311	2.5955	3.0686	2.5392	1.7540
		1,5	2.4466	2.6110	2.8300	2.7571	1.8892
Wufengyou T025	PCs	1,2		0.9606	2.9705	0.6827	1.6154
		1,5		0.6624	1.5311	2.8815	0.7148
Xiangwan 13	PCs	1,2			2.0368	0.2802	1.0224
		1,5			0.8843	2.3718	0.7290
Yaopingxiang	PCs	1,2				2.2980	1.6652
		1,5				1.6452	1.2571
Youyou 122	PCs	1,2					1.1189
		1,5					2.2791

**Table 3.** Classification result of the test set (PC1 and PC2) using PNN (Spread = 4 × 10^−5^).

	**Varieties**	**Classification of PNN Based on PC1 and PC2**						**SUM**

		**P36**	**WT025**	**XW13**	**YPX**	**YY122**	**ZX1**	
Real classification	P36	5	0	0	0	0	0	5
	WT025	0	4	0	0	1	0	5
	XW13	0	0	5	0	0	0	5
	YPX	0	0	0	5	0	0	5
	YY122	0	0	2	0	3	0	5
	ZX1	0	0	0	0	0	5	5
	
	SUM	5	4	7	5	4	5	30

		Classification accuracy = (5 + 4 + 5 + 5 + 3 + 5)/30 = 90%						

Notes: P36 (Pin 36), WT025 (Wufengyou T025), XW13 (Xiangwan 13), YPX (Yaopingxiang), YY122 (Youyou 122), ZX1 (Zhongxiang 1); There are a total of 120 samples for the cross test, 30 samples were randomly selected for the independence test set, and each variety has five samples.

**Table 4.** Classification result of the test set (PC1 and PC5) using PNN (Spread = 4 × 10^−5^).

	**Varieties**	**Classification of PNN Based on PC1 and PC5**						**SUM**

		**P36**	**WT025**	**XW13**	**YPX**	**YY122**	**ZX1**	
Real classification	P36	5	0	0	0	0	0	5
	WT025	0	4	0	0	0	1	5
	XW13	0	0	5	0	0	0	5
	YPX	0	0	0	5	0	0	5
	YY122	0	0	0	0	5	0	5
	ZX1	0	0	0	0	0	5	5
	
	SUM	5	4	5	5	5	6	30
		Classification accuracy = (5 + 4 + 5 + 5 + 5 + 5)/30 = 96.67%						

Note: P36 (Pin 36), WT025 (Wufengyou T025), XW13 (Xiangwan 13), YPX (Yaopingxiang), YY122 (Youyou 122), ZX1 (Zhongxiang 1); There are a total of 120 samples for the cross test, 30 samples were randomly selected for the independence test set, and each variety has five samples.
